# Comparative Pharmacokinetics of Subcutaneous Tolfenamic Acid Under Two Different Anesthetic Protocols in Cats Undergoing Ovariohysterectomy

**DOI:** 10.3390/ani16203257

**Published:** 2026-10-16

**Authors:** Fatma Akin, Orhan Corum, Pedro Marín, Oznur Yılmaz Koc, Devran Coskun, Duygu Durna Corum, Ali Unver, Hatice Rumeysa Ceyhan, María Teresa Yuste, Elena Badillo, Kamil Uney, Muammer Elmas

**Affiliations:** 1 Department of Pharmacology and Toxicology, Faculty of Veterinary Medicine, University of Siirt, Siirt 56100, Türkiye; fatma.akin@siirt.edu.tr (F.A.);; 2 Department of Pharmacology and Toxicology, Faculty of Veterinary Medicine, University of Hatay Mustafa Kemal, Hatay 31060, Türkiye; 3 Department of Pharmacology, Faculty of Veterinary Medicine, University of Murcia, 30100 Murcia, Spain; mariateresa.yuste1@um.es (M.T.Y.); ebp2@um.es (E.B.); 4 Department of Obstetrics and Gyneacology, Faculty of Veterinary Medicine, University of Siirt, Siirt 56100, Türkiye; 5 Department of Veterinary Medicine, Health Services Vocational School, University of Osmaniye Korkut Ata, Osmaniye 80100, Türkiye; hrumeysaceyhan@osmaniye.edu.tr; 6 Department of Pharmacology and Toxicology, Faculty of Veterinary Medicine, University of Selcuk, Konya 42031, Türkiyemelmas@selcuk.edu.tr (M.E.)

**Keywords:** cat, different anesthesia, pharmacokinetics, ovariohysterectomy, tolfenamic acid

## Abstract

The aim of this research was to determine the effect of different anesthesia protocols on the pharmacokinetics of tolfenamic acid in cats undergoing ovariohysterectomy. The research involved 12 cats that underwent ovariohysterectomy. The cats were randomly divided into two equal groups: one (*n* = 6) receiving xylazine–ketamine and the other (*n* = 6) receiving propofol–isoflurane anesthesia. Tolfenamic acid was administered subcutaneously at a dose of 4 mg/kg 30 min prior to ovariohysterectomy in both groups. The plasma samples were analyzed for tolfenamic acid using high-performance liquid chromatography (HPLC)-UV. The terminal elimination half-life (t_1/2λz_), area under the curve (AUC_0-last_), maximum plasma concentration (C_max_), and time to reach peak plasma concentration (T_max_) values after xylazine–ketamine anesthesia were determined to be 3.30 h, 42.17 h*µg/mL, 6.34 µg/mL, and 2 h, respectively. Tolfenamic acid in the propofol–isoflurane anesthetic group showed shorter t_1/2λz_ and T_max_ than the xylazine–ketamine group but a greater C_max_. However, AUC_0-last_ was similar in both anesthesia groups. These results showed that the pharmacokinetics of tolfenamic acid may vary depending on the anesthesia protocol used in ovariohysterectomy. However, there is a need to evaluate the efficacy of tolfenamic acid in cats under both anesthesia regimens.

## Simple Summary

This study investigated whether the choice of general anesthesia affects how the body processes tolfenamic acid, a nonsteroidal anti-inflammatory drug, in cats undergoing ovariohysterectomy. We compared two standard anesthetic protocols: xylazine–ketamine and propofol–isoflurane. While the total amount of the drug absorbed into the bloodstream (overall systemic exposure) was similar in both groups, the rate of absorption differed significantly. Cats that received propofol–isoflurane anesthesia absorbed the subcutaneous medication more rapidly than those receiving xylazine–ketamine. The main clinical implication is that veterinarians can safely administer the standard subcutaneous dose (4 mg/kg) of tolfenamic acid using either anesthetic protocol. Because the overall systemic exposure remains stable, no dosage adjustments are necessary, ensuring effective and simplified postoperative pain management for feline surgical patients.

## 1. Introduction

Ovariohysterectomy, or sterilization, is a commonly performed surgical operation in small animal practice that involves the removal of the uterus and ovaries [1]. Following this surgery, the levels of hormones that trigger heat in the animal will decrease, and the regular estrus cycle will not continue. Ovariohysterectomy eliminates the possibility of estrus behavior, bleeding, and unwanted pregnancy [2]. It is also a treatment option for conditions such as ovarian and uterine tumors and pyometra [3]. Ovariohysterectomy surgery causes pain and inflammation, and effective treatment is crucial in reducing pain, accelerating recovery, and minimizing unwanted complications [4].

Nonsteroidal anti-inflammatory drugs (NSAIDs) have analgesic, anti-inflammatory, and antipyretic effects, which provide an advantage in practice. The pharmacological effect of these drugs results from inhibiting the production of prostaglandins from arachidonic acid via cyclooxygenase (COX) enzymes [5]. There are two types of COX: COX-1 and COX-2. COX-1 is consistently expressed in most tissues and plays a role in physiological processes such as regulating renal blood flow, protecting the gastrointestinal mucosa, and maintaining homeostasis. Although COX-2 is constantly expressed in the kidney and reproductive system, it is particularly inducible in the inflammatory area and plays a role in inflammatory processes [5,6]. The effects of NSAIDs on COXs are significant in terms of adverse effects, and the adverse effects on the gastrointestinal and renal systems and homeostasis are thought to be primarily due to COX-1 inhibition [4,5]. NSAIDs are commonly used to reduce pain and inflammation caused by ovariohysterectomy [1,7]. However, the number of licensed NSAIDs for use in cats is limited due to concerns about toxicity, cost constraints, and insufficient data [5].

Tolfenamic acid is an NSAID from the fenamate category, exhibiting analgesic, antipyretic, and anti-inflammatory properties along with antiendotoxic, anticancer, Alzheimer’s disease-promoting, and antibacterial activities [8,9]. The effects on COX enzymes differ among animal species; it acts as a selective COX-2 inhibitor in goats, but it demonstrates a non-selective inhibitory effect in calves [10,11]. It exhibits a selective effect on COX-2 enzymes in canines [12,13]. Tolfenamic acid is approved for use in cats in Türkiye, Europe, New Zealand, Canada, and Australia [5]. It is used in cats for fever, postoperative pain, acute and chronic inflammatory conditions, and lower respiratory tract disorders [5,14]. It has been reported to successfully reduce pain caused by ovariohysterectomy surgery in cats, and its effect is similar to other NSAIDs such as meloxicam, carprofen, ketoprofen, and robenacoxib [4,15,16].

The main objective of general anesthesia is to induce unconsciousness in the patient, inhibit the perception of pain, and regulate autonomic reflexes [17]. The most frequent general anesthetic protocols used for ovariohysterectomy surgery in cats are xylazine–ketamine and propofol–isofulurane. Ketamine is a dissociative general anesthetic that provides amnesia, analgesia, and immobilization. However, because it does not provide sufficient muscle relaxation, it is used in combination with xylazine, an α2 adrenoreceptor agonist that has effects such as muscle relaxation, analgesia, sedation, and anxiolysis [18,19]. Isoflurane is a common inhalational anesthetic characterized by its minimal metabolic rate and low blood gas solubility, whereas propofol is a brief-acting intravenous anesthetic frequently employed for the initiation and sustenance of general anesthesia. These two drugs are often used in combination to enhance anesthetic efficacy and reduce side effects [20].

Although there is limited information in the drug prospectus inserts regarding the pharmacokinetics of tolfenamic acid in cats [14], to our knowledge, no detailed pharmacokinetic study has been conducted. The pharmacokinetics of tolfenamic acid have been examined across many animal species, demonstrating notable interspecies variations [6,9,21,22,23,24]. Pharmacokinetics facilitates the quantitative assessment of the processes involved in the absorption, distribution, metabolism, and excretion of medicines [25]. A comprehensive understanding of these pharmacokinetic parameters in the target species is crucial for determining correct dosage strategies in clinical practice [26]. Furthermore, in the diseases or conditions for which the drug use is recommended, conducting pharmacokinetic studies of the drugs is critical for effective treatment [26]. Despite the effective application of tolfenamic acid in cats’ ovariohysterectomy procedures, there is a lack of data regarding its pharmacokinetics. It has been reported that anesthetic drugs can alter the pharmacokinetics of other drugs used concurrently during surgical procedures [27]. The effects of anesthetic drugs on physiological parameters vary [28], and therefore, changes in the pharmacokinetics of the drug may differ depending on the anesthesia protocol. This study hypothesized that the distinct hemodynamic and physiological effects of xylazine–ketamine versus propofol–isoflurane anesthesia induce significant alterations in the absorption and disposition kinetics of subcutaneously administered tolfenamic acid in cats undergoing ovariohysterectomy. The aim of this study was to determine the pharmacokinetics of tolfenamic acid administered subcutaneously at a dose of 4 mg/kg during ovariohysterectomy performed under xylazine–ketamine and propofol–isoflurane anesthesia in cats.

## 2. Materials and Methods

### 2.1. Chemicals

A tolfenamic acid analytical standard with a purity of at least 98% was purchased from Sigma Aldrich (St. Louis, MO, USA). Chemicals used for the analysis of this drug were HPLC grade. Acetonitrile and methanol were supplied by Chemlab (Zedelgem, Belgium) and orthophosphoric acid by Sigma Aldrich (St. Louis, MO, USA). Parenteral (Tolfenak Injection Solution, 40 mg/mL, Alke, Istanbul, Türkiye) formulation of tolfenamic acid was used to administer the drug to cats. Anesthesia was induced with commercial preparations of xylazine (Xylazin Bio 2%; Bioveta, Ivanovice na Hané, Czech Republic)–ketamine (Ketasol 10%, VetViva Richter GmbH, Wels, Austria) and propofol (Propofol-Pf Mct 1% Injection/Infusion Emulsion; Polifarma, Tekirdağ, Türkiye)–isoflurane (Vetflurane, Virbac, Carros, France) anesthesia.

### 2.2. Animals

Twelve healthy female domestic cats (2–4 years old; body weight 2.0–3.4 kg) were used in this study. The cats were selected from those brought to the Obstetrics and Gynecology Department of Siirt University Animal Health Application and Research Hospital for scheduled ovariohysterectomy. Cats that were 6–10 weeks post-estrus, clinically healthy on physical examination, and had normal findings in hematological analysis, serum biochemistry (liver and kidney function tests), vaginal cytology, and abdominal ultrasonography were included in the study. Exclusion criteria for cats in the study included a history of chronic disease, liver or kidney failure, hematological abnormalities, pregnancy, lactation, contraindications to anesthesia, and use of NSAIDs or corticosteroids within the last 30 days. Cat owners were informed about the anesthesia procedures and the study, and written informed consent was obtained. Cats were hospitalized one day before surgery and fasted for 12 h prior to the procedure. The study was approved (protocol number: 2024/07-47) by the Local Ethics Committee of Siirt University.

### 2.3. Experimental Design

Cats were randomly allocated into two equal groups (*n* = 6 per group) using computer-generated randomisation: Group 1 received xylazine–ketamine anesthesia, and Group 2 received propofol–isoflurane anesthesia. A single surgeon performed all ovariohysterectomy procedures via a standard ventral midline approach within one month.

Group 1 (X/K): First, tolfenamic acid was administered to the cats subcutaneously at a dose of 4 mg/kg. For anesthesia induction, xylazine was administered intramuscularly at a dose of 1 mg/kg ten minutes after the tolfenamic acid administration, followed ten minutes later by ketamine at a dose of 10 mg/kg. The surgical procedure commenced ten minutes after the ketamine administration. Thus, tolfenamic acid was administered 30 min prior to the surgical procedure.

Group 2 (P/I): First, tolfenamic acid was administered to the cats subcutaneously at a dose of 4 mg/kg. For anesthesia induction, propofol was administered intravenously at a dose of 6 mg/kg fifteen minutes after tolfenamic acid administration. For anesthesia maintenance, 2–3% isoflurane in oxygen was administered via a semi-closed rebreathing system following endotracheal intubation 10 min after propofol administration. The surgical procedure began approximately 5 min after isoflurane anesthesia. Therefore, tolfenamic acid was administered 30 min before the surgical procedure.

### 2.4. Surgical Procedure

Palpebral and pedal reflex checks were performed, and induction was considered complete when the cat was fully relaxed and had lost lower mandibular tone. Following the confirmation of normal aseptic surgical protocols, ovariohysterectomy was performed on all cats via ventral midline laparotomy. The ovarian stalks and uterine body were ligated with absorbable suture material (polyglactin 910) using a modified Miller knot technique. The duration of the surgery, from skin incision to skin closure, was recorded as 40–45 min. During the procedures, each cat received approximately 50–75 mL of isotonic solution for fluid support. Postoperative pain and recovery were clinically assessed by the attending veterinarians (O.Y.K. and A.U.) as part of routine care. Rescue analgesia was accessible if necessary; yet, no cat needed rescue analgesia.

### 2.5. Drug Administration and Blood Sampling

Tolfenamic acid was administered subcutaneously (between the shoulder blades) at a dose of 4 mg/kg to both groups 30 min before the surgical procedure began. To collect blood samples, a 22-gauge catheter was aseptically placed in the antebrachial vein under sedation one day prior to surgery. Blood samples were collected into lithium heparin tubes at 0 (control, before tolfenamic acid administration), 15, 30 and 45 min, and 1, 2, 4, 6, 8, 10, 12, 24, and 48 h after drug administration. The volume of blood drawn was adjusted for each cat, ensuring that no more than 10% of the total blood volume was removed during the research duration (maximum of 0.6–1 mL each sample). Following each collection, the catheter was flushed with 0.6–1 mL of heparinised saline. The heparin tubes were mixed by inversion and then centrifuged (at 4000× *g* for 10 min) within 1 h. After centrifugation, plasma samples were transferred to microcentrifuge tubes and then stored at −80 °C until analysis.

### 2.6. Tolfenamic Acid Analysis

Plasma tolfenamic acid concentrations were determined by high-performance liquid chromatography (HPLC) with ultraviolet (UV) detection using a previously validated method with minor modifications [21]. Briefly, to denature the proteins, 150 µL of acetonitrile was added to 100 µL of plasma sample. The mixture was vortexed for 30 s and centrifuged at 12,000× *g* for 10 min. The supernatant was transferred to autosampler vials, and an aliquot (20 µL) was injected into the HPLC system (Shimadzu, Kyoto, Japan). Chromatographic separation of tolfenamic acid was performed using an InertSustain C18 column (250 × 4.6 mm inner diameter, 5 μm particle size, GL Sciences, Tokyo, Japan) maintained at 40 °C and a wavelength of 289 nm. The mobile phase consisted of acetonitrile and 0.1% orthophosphoric acid (65:35 *v*/*v*%), with a flow rate of 1 mL/min. The data were processed using the LC Solution software program (Version 1.25 SP5).

### 2.7. Validation of the Analytical Method

Linearity, limits of detection (LOD) and quantification (LOQ), recovery, and intra-day and inter-day accuracy of the HPLC method were verified in plasma according to the European Medicines Agency guidelines [29]. Linearity was assessed by linear regression analysis of calibration curves constructed using control plasma samples spiked with tolfenamic acid at concentrations from 0.04 to 20 μg/mL. The LOD was defined as the plasma concentration of tolfenamic acid that produces a signal-to-noise ratio of 3, and the LOQ was determined as the plasma concentration of tolfenamic acid that produces a signal-to-noise ratio of 6. Plasma spiked with tolfenamic acid at three different concentrations (0.1, 1 and 10 μg/mL) was analyzed three times on five separate days for assessing the recovery and intraday and interday precision. These concentrations were then compared to working standards to determine recovery. Precision was calculated and expressed as coefficient of variation (CV%).

### 2.8. Pharmacokinetic Analysis

Pharmacokinetic parameters were obtained from plasma concentrations of tolfenamic acid in each cat by a non-compartmental assay via WinNonlin 6.1.0.173 software (Pharsight; Certara, St. Louis, MO, USA). The terminal elimination half-life (t_1/2λz_) was determined by formulating t_1/2λz_ = ln(2)/λ_z_. Area under the curve (AUC) was measured by linear up/log down method. The mean residence time (MRT), apparent volume of distribution (V_darea_/F), and apparent total body clearance (Cl_T_/F) were calculated. AUC extrapolated from the last measurable concentration to infinity as a percentage of total AUC (AUC_extrap_%) was determined. The peak plasma concentration (C_max_) and the time to reach C_max_ (T_max_) were determined directly from the observed data.

### 2.9. Statistical Analysis

Statistical analysis was performed using SPSS 22.0 software (IBM Corp., Armonk, NY, USA). A *p* value of <0.05 was considered statistically significant. Pharmacokinetic parameters are reported as geometric mean (min-max), while T_max_ is reported as median (min-max). Shapiro–Wilk test was used to verify normal distribution of the pharmacokinetic parameters. Independent *t* test was performed to analyze statistically significant differences between treatment groups.

## 3. Results

### 3.1. Validation of Analytical Methods

The method has good specificity without any peak in chromatogram at retention time of tolfenamic acid. The linearity of working and calibration curves was excellent in the concentration range of 0.04–20 μg/mL with R^2^ values > 0.999. The LOD was 0.02 μg/mL and the LOQ was 0.04 μg/mL. The extraction procedure was highly efficient and the mean recovery ratio of tolfenamic acid was above 95%. The coefficients of variation for intra- and inter-day were <6.88% and <5.49%, respectively. The intra- and inter-day bias were ±4.15% and ±3.23%, respectively. Overall, the validated approach has demonstrated to be reliable for measuring tolfenamic acid in cats’ plasma using HPLC.

### 3.2. Pharmacokinetics

**Figure 1:**
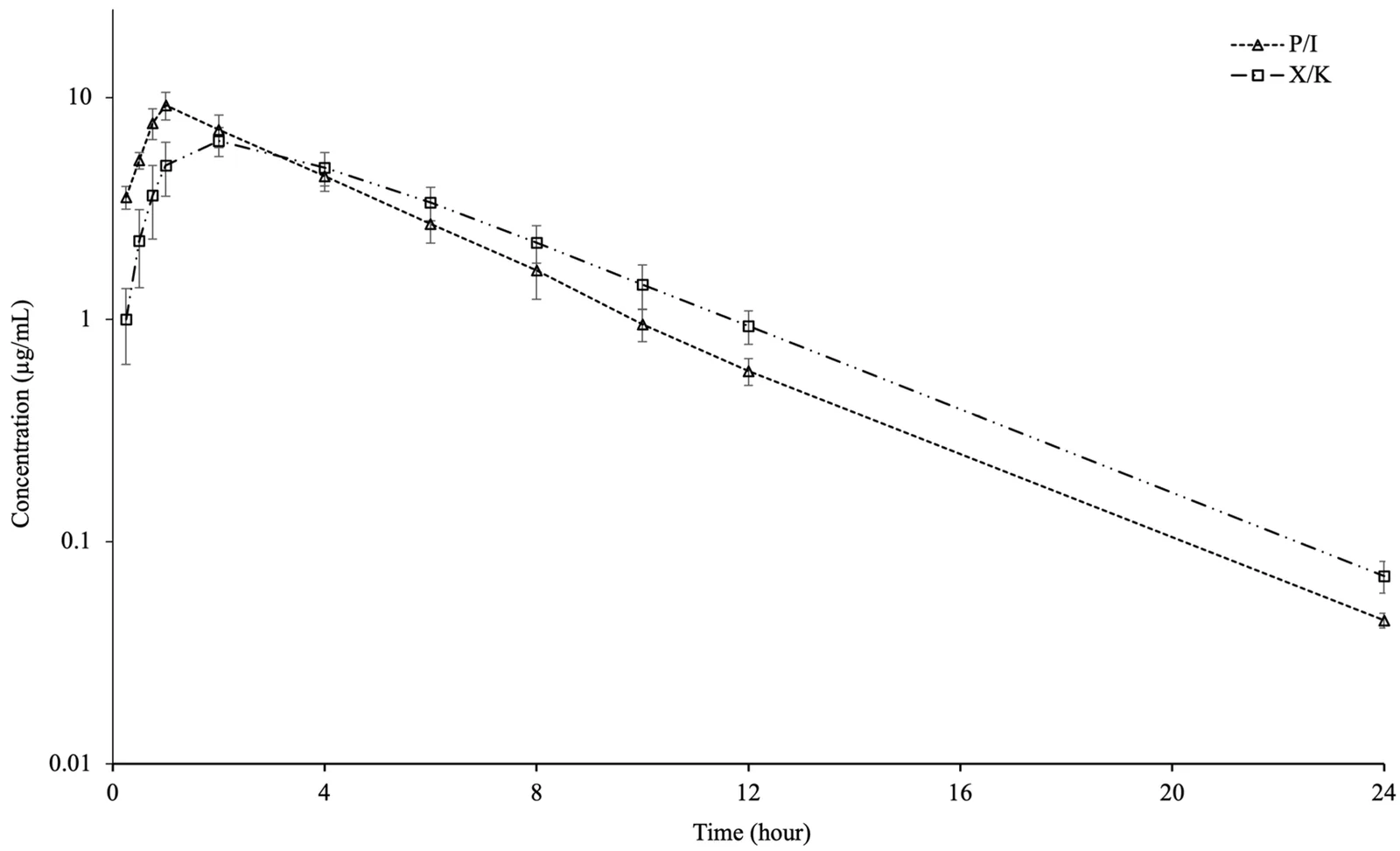
Semi-logarithmic plasma concentration–time curves following subcutaneous administration of tolfenamic acid at a single dose of 4 mg/kg in cats undergoing ovariohysterectomy under xylazine–ketamine (X/K) and propofol–isoflurane (P/I) anesthesia (mean ± SD, *n* = 6).

The semi-logarithmic plasma concentration–time curve following subcutaneous administration of tolfenamic acid at a dose of 4 mg/kg in cats undergoing ovariohysterectomy with different anesthesia protocols is presented in Figure 1. Tolfenamic acid was detected up to 24 h in both anesthesia groups. Table 1 presents non-compartmental pharmacokinetic parameters. The t_1/2λz_ and MRT_0-∞_ of tolfenamic acid were longer in cats undergoing ovariohysterectomy that received xylazine–ketamine anesthesia compared to those that received propofol–isoflurane anesthesia. Cats administered propofol–isoflurane exhibited a greater C_max_ and a shorter T_max_ than those receiving xylazine–ketamine anesthesia (*p* < 0.05). The AUC, V_darea_/F, and Cl_T_/F were comparable in both anesthesia groups (*p* > 0.05).

## 4. Discussion

This study revealed for the first time the pharmacokinetics of tolfenamic acid in cats undergoing ovariohysterectomy under xylazine–ketamine and propofol–isoflurane anesthesia. The study concluded that pharmacokinetic parameters of tolfenamic acid, such as t_1/2λz_, C_max_, and T_max_, varied depending on the anesthesia protocol.

Overall, glucuronidation metabolism plays a crucial role in the elimination of NSAIDs. The distribution of these medications in the cat varies from that in the dog due to the low capacity of hepatic glucuronidation in the cat [5]. It has been reported that the t_1/2λz_ of NSAIDs, especially those metabolized via glucuronidation reactions, is longer in cats than in dogs [5]. Therefore, there is a significant potential for NSAID toxicity in cats, and using this group of drugs at the appropriate dose and dosing interval is crucial for safety [30,31]. The adverse effects of NSAIDs are particularly related to the gastrointestinal, renal, and hepatic systems [5]. Tolfenamic acid has a lower risk of gastrointestinal damage than many other NSAIDs and is well tolerated at therapeutic doses [32,33,34]. Similarly, oral administration of tolfenamic acid at a dose of 4 mg/kg for 14 days has no adverse effects on renal function in cats [35]. The tolfenamic acid at a dosage of 4 mg/kg for 3 days did not induce any change in diarrhea, vomiting, polyuria, polydipsia, or aggression in the cats [5].

The recommended dose of tolfenamic acid in cats is 4 mg/kg via oral or subcutaneous route [5,14]. Tolfenamic acid was administered subcutaneously at a dosage of 4 mg/kg to cats getting ovariohysterectomy and showed efficacy [15,16]. For ovariohysterectomy procedures in cats, a single dose of NSAID injection is frequently suggested [7]. Preoperative analgesic therapy is recommended because it can either lead to higher plasma and tissue fluid/inflammatory exudate levels or reduce central changes by decreasing the amount of noxious information generated peripherally [36,37]. Therefore, in this study, a single dose of 4 mg/kg tolfenamic acid was administered subcutaneously 30 min before ovariohysterectomy.

Physiological alterations occur in the body due to surgical procedures and anesthetic agents. Surgery causes stress associated with pain, anxiety, cardiovascular instability, and hemodynamic changes, triggering the release of catecholamines, corticosteroids, and cytokines, and in turn initiating a complex series of metabolic events that can last for several days [38]. Anesthetic drugs alter body perfusion, hepatic and metabolic functions, renal functions, and the binding of drugs to plasma proteins [27]. Therefore, surgical procedures may cause differences in the pharmacokinetics of concomitantly used drugs [39]. Ovariohysterectomy surgery altered the disposition of meloxicam and delayed the elimination of carprofen in dogs [36,40].

The t_1/2λz_ is defined as the time it takes for a drug’s concentration in the body to decrease to half of its initial amount [41]. The t_1/2λz_ of tolfenamic acid in cats undergoing ovariohysterectomy was longer in the xylazine–ketamine than in the propofol–isoflurane anesthesia groups. However, the difference between the two anesthetic groups was only 11%, which may be clinically insignificant. The t_1/2λz_ is a parameter derived from both clearance and volume of distribution and is affected by changes in these parameters [42]. The V_darea_/F and Cl_T_/F values were comparable in both anesthesia groups in this study. Since the absorption process will not have an impact, intravenous administration is the most suitable route for determining these two parameters. In this study, since tolfenamic acid was administered solely via the subcutaneous route, variations in absorption across the different anesthesia groups could not be determined. Although these two parameters were similar in both anesthesia groups, the change at t_1/2λz_ may be due to differences in absorption between the anesthesia groups.

The V_darea_/F and Cl_T_/F of tolfenamic acid were similar in cats undergoing ovariohysterectomy under xylazine–ketamine and propofol–isoflurane anesthesia. NSAIDs generally have a limited volume of distribution owing to their significant binding to plasma proteins and their ionized condition in the bloodstream. Fenamate derivatives such as tolfenamic acid have pKa values of approximately 4 and are largely ionized at physiological pH levels (blood = 7.4) [43]. Tolfenamic acid has a high binding to plasma proteins in a variety of animal species, including the dog [21]. Therefore, tolfenamic acid generally has a low volume of distribution [44]. Tolfenamic acid undergoes hepatic metabolism and is eliminated via urine and bile, along with its metabolites [45,46]. It has been reported that it is mostly excreted unchanged in cats [14]. Surgical procedures and anesthetics have been reported to affect cardiac output, tissue perfusion, renal and hepatic blood flow, biotransformation enzyme activities, and plasma protein levels, and this may affect the volume of drug distribution and clearance [27,39,47]. Since there was no control group that did not undergo surgery in this study, the effect of anesthesia protocols on this could not be evaluated. However, in dogs undergoing ovariohysterectomy procedure, the V_d_ value of meloxicam decreased after IV administration, while the Cl value remained unchanged [40]. The effects of xylazine–ketamine and isoflurane anesthesia on hemodynamics and cardiac function [28], the effects of xylazine–ketamine and ketamine-propofol anesthesia on pulse rate, rectal temperature, and hematobiochemical profile [48], and the effects of medetomidine, xylazine–ketamine–halothane, and propofol on glomerular filtration differed [49]. These results show that the pathophysiological effect differs according to the anesthesia protocol. To our knowledge, there are no studies comparing the pathophysiological effects of xylazine–ketamine and propofol–isoflurane anesthesia protocols; however, since these two anesthesia protocols exhibit similar effects, the V_darea_/F and Cl_T_/F may be similar in these groups.

The C_max_ of tolfenamic acid was higher and the T_max_ shorter in cats treated with propofol–isoflurane than in animals treated with xylazine–ketamine anesthesia. This shows that tolfenamic acid reaches higher concentrations in the blood in a shorter time after propofol–isoflurane anesthesia. The C_max_ indicates the rate and extent of absorption from the injection site of the drug according to the elimination rate [50]. The rate and extent of drug absorption can vary depending on the drug formulation, injection site, and tissue perfusion. Both groups were used with the same formulation of tolfenamic acid, and the injections were administered in the same area. Therefore, changes in these parameters may result particularly from changes in tissue perfusion. During surgical procedures, decreased cardiac output and peripheral perfusion reduce the absorption of drugs from the injection site, which can lead to a decrease in C_max_ and a prolongation of T_max_ [27]. The effects of anesthetic agents on hemodynamics and cardiac function vary [28]. Xylazine–ketamine anesthesia caused more changes in hemodynamics, cardiac function, and glucose and lipid metabolism compared to isoflurane anesthesia [28]. Heart rate decreased significantly 15 min after xylazine–ketamine anesthesia but was similar to that of isoflurane at 40 min [28]. In this study, the differences in C_max_ and T_max_ values of tolfenamic acid may be due to differences in hemodynamics and cardiac functions among the anesthesia groups. However, it should be noted that this assessment is merely an interpretation, as the effects of anesthetics on hemodynamics and cardiac functions were not evaluated in this study.

The AUC of tolfenamic acid was similar in cats undergoing ovariohysterectomy under xylazine–ketamine and propofol–isoflurane anesthesia. Tolfenamic acid was detected up to 24 h in both anesthesia groups. AUC represents the total systemic exposure to a drug over time, and systemic clearance and bioavailability play a crucial role in determining AUC (Dose × F/Cl) [51,52]. The similarity of AUC in both anesthesia groups may be due to the relationship between systemic clearance and bioavailability. The fact that the AUC value was similar in both anesthesia groups may indicate that the systemic exposure to tolfenamic acid is similar and that it can be used with two different anesthesia protocols.

To the best of our knowledge, pharmacodynamic studies regarding the dose-concentration relationship of tolfenamic acid in cats are insufficient; therefore, the relationship between plasma concentration and pharmacodynamic efficacy could not be compared. In addition, it has been reported that plasma concentrations of NSAIDs do not reflect their effectiveness [53]. Plasma proteins tend to accumulate in the inflammatory region, and drugs that bind highly to these proteins, such as tolfenamic acid, can accumulate in this region and exert a longer-lasting effect [10]. Therefore, the dose–response relationship of tolfenamic acid in cats undergoing ovariohysterectomy needs to be established.

The findings of this study should be interpreted in light of certain limitations. First, the relatively small number of animals (*n* = 6) used in each group may limit the broader generalization of the pharmacokinetic results. Second, because there was no non-surgical control group, the absolute effects of the surgical procedure and anesthesia protocols on the pharmacokinetics of tolfenamic acid from a baseline state could not be evaluated. Third, although the alterations in the absorption phase were attributed to hemodynamic changes, specific cardiovascular parameters such as cardiac output or blood pressure were not recorded during the surgeries. Fourth, since NSAIDs—including tolfenamic acid—tend to accumulate in exudate fluid, plasma concentrations may not reflect the therapeutic effect. Therefore, studies characterizing the exudate concentration and pharmacokinetics of tolfenamic acid in cats are needed to evaluate its therapeutic effect. Fifth, although postoperative pain and the recovery process were clinically assessed, no feline pain scoring system was employed. Therefore, the inability to fully rule out confounding effects associated with pain—and the inability to assess the relationship between pain severity and the biomarker—represents a limitation. Additionally, the plasma protein binding ratio and the metabolism of tolfenamic acid were not determined in cats undergoing ovariohysterectomy, which could have provided deeper mechanistic insights. Finally, as this research focused exclusively on pharmacokinetics, the clinical efficacy and dose–response relationship of tolfenamic acid under these anesthetic protocols remain to be evaluated in future studies.

## 5. Conclusions

This study demonstrates for the first time the pharmacokinetic profile of subcutaneously administered tolfenamic acid in cats undergoing ovariohysterectomy under different anesthesia protocols. Although propofol–isoflurane anesthesia resulted in statistically significant alterations in the absorption phase—specifically a higher peak C_max_ and a shorter time to reach peak concentration T_max_—these variations are unlikely to be of clinical significance. The comparable overall systemic exposure (AUC), apparent clearance, and relatively minor difference in the t_1/2λz_ between the groups indicate that tolfenamic acid can be safely and effectively administered at the standard 4 mg/kg dose regardless of the chosen anesthetic regimen, without the need for dosage adjustments. Nevertheless, to fully optimize postoperative pain management in feline practice, future studies should investigate the pharmacodynamic efficacy, dose–response relationships, and target tissue accumulation of tolfenamic acid in surgical patients. Furthermore, this study did not include a group of cats that received either anesthesia regimen without undergoing a surgical procedure. Consequently, it is difficult to distinguish whether the observed alteration in the pharmacokinetics of tolfenamic acid resulted from surgical trauma/stress or the effects of the anesthetic agents. Therefore, further studies incorporating anesthetic control groups are needed.

## Figures and Tables

**Table 1:** Pharmacokinetic parameters of tolfenamic acid expressed following subcutaneous administration of tolfenamic acid at a single dose of 4 mg/kg in cats undergoing ovariohysterectomy under xylazine–ketamine (X/K) and propofol–isoflurane (P/I) anesthesia (*n* = 6).

Parameter	*p* Values	95% Confidence Intervals (Lower to Upper)	X/K Anesthesia	P/I Anesthesia
t_1/2λz_ (h)	0.04	−0.52 to 0.14	3.30 (3.11–3.56)	2.97 (2.84–3.11) *
MRT_0-∞_ (h)	<0.001	−1.55 to 0.99	5.85 (5.45–6.17)	4.59 (4.38–4.79) *
AUC_0-last_ (h*µg/mL)	0.981	−8.42 to 8.24	42.17 (35.35–53.21)	42.16 (33.02–46.92)
AUC_0-∞_ (h*µg/mL)	0.951	−8.61 to 8.14	42.51 (35.74–53.54)	42.36 (33.22–47.09)
AUC_extrap_ (%)	-	-	0.78 (0.61–1.10)	0.45 (0.35–0.61)
C_max_ (µg/mL)	0.02	1.37 to 4.32	6.34 (5.37–6.80)	9.16 (7.24–10.37) *
T_max_ (h)	0.01	−1.51 to 0.66	2 (1.00–2.00)	0.75 (0.75–0.75) *
V_darea_/F (L/kg)	0.359	−144.99 to 57.59	0.45 (0.34–0.57)	0.41 (0.35–0.54)
Cl_T_/F (L/h/kg)	0.975	−18.56 to 19.11	0.09 (0.07–0.11)	0.09 (0.08–0.12)

*: This shows a statistically significant difference compared to xylazine–ketamine anesthesia (*p* < 0.05). t_1/2λz_: terminal elimination half-life, MRT: mean residence time, AUC: area under the concentration–time curve, AUC_extrap_%: area under the plasma concentration–time curve extrapolated from t_last_ to ∞ in % of the total AUC, C_max_: peak plasma concentration, T_max_: time to reach peak concentration, V_darea_/F: apparent volume of distribution, and Cl_T_/F: apparent total body clearance.

## Data Availability

The data presented in this study are available upon request from the corresponding authors.
